# Research on process parameter optimization of irregular coal face

**DOI:** 10.1038/s41598-024-62517-x

**Published:** 2024-05-27

**Authors:** Qinghai Li, Zijun Wang, Nan Liu, Haoran Zhang, Zhaoying Li

**Affiliations:** College of Energy and Mining Engineering, Shandong University of Science and Technology, Qingdao, 266590 People’s Republic of China

**Keywords:** Pressure bumping, Roof cutting pressure relief, Mining speed, Stopping line position, Coal, Civil engineering

## Abstract

Based on the engineering background of 1353 working face in Daizhuang Coalmine, the paper identifies three primary issues with the working face mining process: conventional pressure relief means are limited, risk of impact and the length of working face changes, It also proposes comprehensive control measures to improve the blasting roof cutting scheme, optimize mining speed and the location of the stopping line. The three improvement measures mentioned above are simulated numerically, and the effects of the drilling and blasting plan, mining speed, and stopping line location on stress distribution are determined. The results show that by implementing the three improvement measures, the stress variation interval can be efficiently controlled and the working face's production safety can be increased. Finally, it is determined that the 1353 working face of Daizhuang Coalmine adopts the pressure relief method of drilling on one side and cutting the roof 20 m deep on the other side, and the mining speed of the working face is 3 m/day and the length of the stopping line is 85 m. Based on the on-site monitoring results, the implementation of comprehensive treatment measures can effectively improve the surrounding rock state of 1353 working face, which has certain guiding significance for the mining of irregular working face.

## Introduction

With the progressive deepening of mining operations, the incidence of regional pressure bumping events has become more frequent, particularly in areas with intricate geological structures^1,2^. In view of the prevention of such mine disasters, different experts and scholars have given different solutions, among which roof cutting and pressure releasing is a common way^3–6^. In the transverse direction, the roof cutting measures can effectively destroy the integrity of the rock strata, cut off the transmission of force, reduce the range of rock strata moving in the transverse direction at the same time, and achieve the purpose of reducing the intensity of mine pressure. In the longitudinal direction, the roof cutting measures can promote the timely collapse of the roof in the goaf, reduce the range of suspended roof and reduce the impact risk of large-scale suspended roof fracture^7–10^. Research conducted by Gao^11^ has demonstrated that the energy generated during blasting can induce significant loosening of the rock mass, promoting thorough and timely collapse of the goaf's roof. This process aids in the redistribution of stress across the roof of the goaf, enhancing the stability of the surrounding rock in the roadway. Zhao^12^ changed the roof structure into a short cantilever beam structure by pre-splitting cutting, which reduced the stress concentration of the coal pillar on the side of the roadway, and verified the feasibility of roof cutting and pressure releasing under complex geological conditions. He^13,14^ formed a model of the 'short cantilever beam' by directional roof cutting, released the stress at the roof, and filled the goaf by using the expansion characteristics of the caving rock. The above scholars explained the mechanism and principle of blasting roof cutting and pressure relief through corresponding research. Building upon this foundation, Wang^15^ compared the influence of different influencing factors on the stress distribution of roadway in roof cutting and pressure releasing, and pointed out that the height of roof failure had a great influence on the pressure releasing effect. In addition to roof cutting and pressure releasing, controlling the mining speed of working face can also effectively reduce the risk of pressure bumping. Yang^16,17^ believes that the influence of working face mining speed on rock burst is essentially the result of the interaction between different loading rates and rock deformation rates. High loading rates increase the tensile strength of rock, the loading rate of maximum principal stress and the unloading rate of minimum principal stress in the coal body in front of the coal wall, and the strain energy density of shallow coal body, which leads to the increase of the risk and damage degree of pressure bumping in surrounding rock. Liu^18^ and Gao^19^ used FLAC numerical simulation to study the law of mine pressure behavior under different mining speeds, which provided a strong basis for the calculation of support resistance in fully mechanized mining face. Based on the actual situation of the site, Xie Guangxiang^20–22^ explored the influence of the advancing speed of the working face on the stress of the surrounding rock of the roadway through FLAC2D, and concluded that the appropriate mining speed was helpful to the stability of the roadway. Han^23^ established a time–space subsidence model of overburden movement, which provides a reference for early warning of mine pressure control from the perspective of roof subsidence. The above-mentioned authors have studied the mining speed through the influencing mechanism, research methods and field application. At present, most studies focus on the prevention of rockburst unilaterally. However, in the face of irregular working faces under complex geological conditions, a single pressure relief method is not enough to solve the problem of rockburst. Therefore, this paper closely fits the actual situation of the site, comprehensively controls rock burst from multiple angles, and provides reference for the mining of complex and irregular working faces.

On the basis of previous studies, this paper takes 1353 irregular working face of Daizhuang Coalmine as the engineering background, analyzes the main problems existing in the mining process, and determines the reasonable process parameters through numerical simulation method, so as to provide reference for on-site safety production.

## Engineering overview and risk factors analysis

The Daizhuang Coalmine is located 6 km north of Jining City, Shandong Province, China, with an annual output of 3.2 million tons of coal. The No. 3 coal seam, which is part of the nearly horizontal coal seam and has an average buried depth of 580.58 m, is currently the primary mining focus. The general situation of its top and bottom plates is shown in Table 1. No. 3 coal seam is divided into upper and lower parts. The upper coal seam is 5.3 m thick and the lower coal seam is 3.3 m thick, with siltstone with an average thickness of 3.3 m in the middle, in which the upper coal seam of No. 3 coal seam (hereinafter referred to as No. 3 coal seam) is mainly mined.

**Table 1:** General situation of roof and floor of No.3 coal seam.

Location	Rock type	Thickness (m)	Lithologic character
Basic top	Silty sandstone interbed	10.37–23 17.08	Gray to light gray, with sandstone bands in some areas. The composition is mainly quartz, followed by feldspar, containing a small amount of dark minerals, and the bottom contains a large number of plant debris fossils, with high argillaceous content and hardness of 4–5
Direct roof	Argillaceous siltstone	0.6–1.3 1.2	Gray–black, aluminum-bearing, dense and massive, with broken rocks, undeveloped bedding and developed joints, with hardness of 4
Direct bottom	Siltstone	1.2–5.88 3.11	Light gray, dense and hard, argillaceous cementation, no obvious bedding, developed joints and hardness of 4
Basic bottom	Silty sandstone interbed	14.01–20.4 16.65	Gray to light gray, locally sandwiched with sandstone bands, mainly composed of quartz, followed by feldspar, containing a small amount of dark minerals, with hardness of 4–5

**Figure 1:**
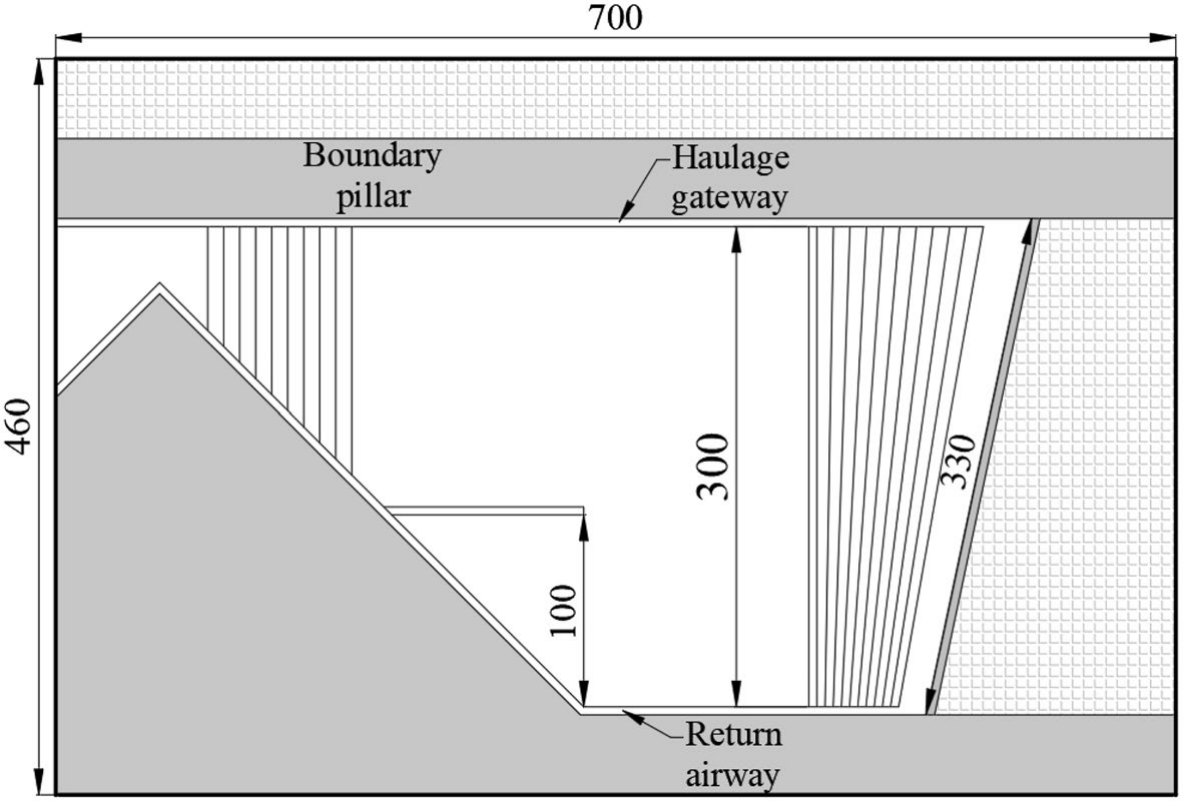
1353 working face layout.

Adjacent to the boundary pillar of the eastern mining area, The No. 3 coal seam's 1353 working face is mined to its full height in a single session using retreating mining technology and the longwall mining method, The free caving method is used to treat goaf. The 1353 working face was shaped like a typical "knife handle" irregular shape due to the prevailing geological conditions. In the early stage of mining, the width of the working face is 330 m. After mining 215 m along the return airway, the width of the working face is reduced to 200 m. After the working face continues to mine 140 m, the working face begins to shrink that the haulage gateway gradually approach the return airway (Fig. 1).

In contrast to the conventional working face, the spatial structure of overburden rock in irregular working face is more complex, the failure form of surrounding rock is more irregular, the stress evolution process is more complex and changeable, and the failure law and time-effect characteristics of surrounding rock are more complex. To guarantee the safe manufacturing of 1353 irregular working face, it is important to examine the elements that lead to disasters and provide appropriate preventive measures. The primary causes of disasters are as follows:

1. Conventional pressure relief means are limited
  The side of 1353 working face haulage gateway is boundary pillar. According to the 'Coal Mine Water Control Rules' provisions, the waterproof coal pillar must be left at the boundary of the adjacent mine, and it is strictly prohibited to excavate in various water-proof coal pillars. Therefore, the pressure relief holes are strictly prohibited in the boundary pillar of the mine field, and only drill pressure relief holes in the side of the haulage gateway, which does not meet the requirements of pressure bumping prevention and control.
2. Risk of impact
  The hard sandstone above the 1353 working face has strong stability, which makes the overburden rock not easy to collapse. However, when the suspension length of the roof reaches the maximum value that the overburden can be self-stabilized, the overburden will break, and the large-scale caving overburden will cause strong impact in the goaf, resulting in serious impact accidents on the working face. Furthermore, the pillar experiences a greater amount of static and dynamic load during the mining stage. This makes it more vulnerable to damage from mining stress, which can result in pressure bumping.
3. The length of working face changes
  Limited to geological conditions, the length of 1353 working face changes many times in the process of advancing. As the width of the working face decreases, the strike breaking step distance will increase, and the tendency breaking distance will decrease, which will cause the integrity of the overlying roof structure to be damaged. These areas are often prone to high-energy mine earthquakes, which increases the risk of working face mining.

## Numerical simulation of roof cutting pressure relief

The impact risk assessment indicates that pressure relief measures should be implemented for the medium impact risk of belt crossheading in the 1353 working face mining process. Aiming at the problem that the pressure relief hole cannot be drilled on the boundary pillar, it is proposed to replace the pressure relief hole with the method of roof cutting and pressure relief on the boundary pillar side of the haulage gateway. The FLAC3D numerical simulation method is used to analyze the effectiveness of the roof cutting and pressure releasing, and the specific parameters of the roof cutting are determined.

**Figure 2:**
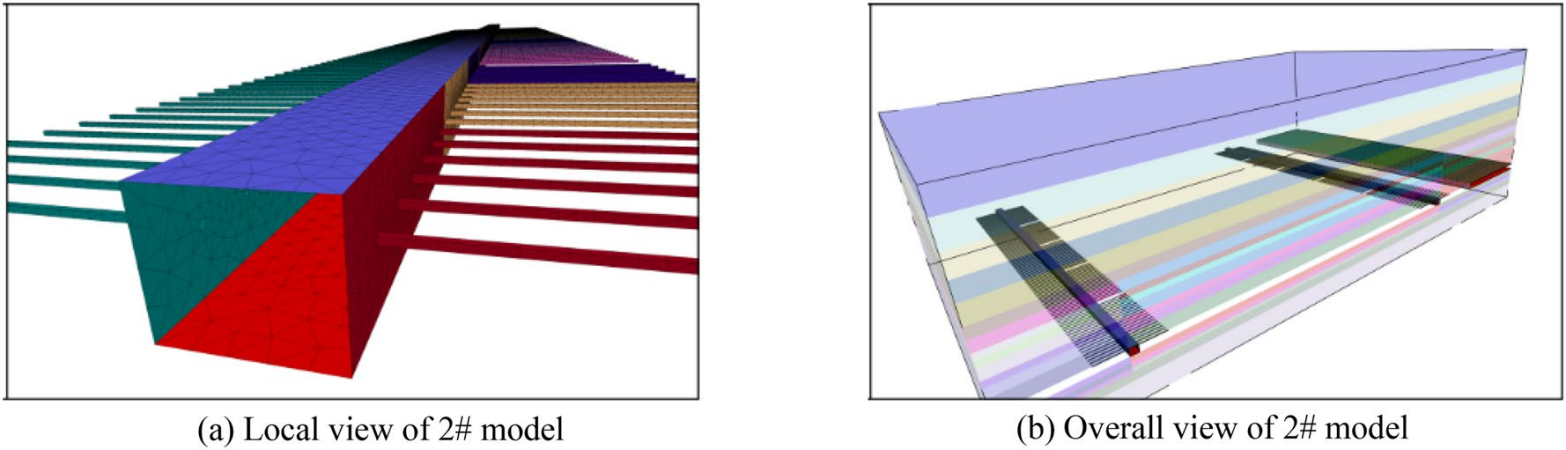
Diagram of 2# model.

Establish a numerical model with the 1353 working face as the background, and the specific rock parameters are shown in Table 2. On the basis of simulating the actual rock stratum size conditions as much as possible, the calculation speed and calculation cost are taken into account. The model is set to be 500 m × 300 m × 147 m (length × width × height) as shown in Fig. 2. The Mohr–Coulomb model is adopted. The bottom boundary of the model is fixed, the normal displacement of the front and rear left and right boundaries is fixed, the top boundary is free, and the buried depth of the 1353 working face is about 600 m. According to the calculation of the height of the overlying strata, the fixed uniform load of 10.46 MPa is applied to the top of the model.

**Table 2:** Mechanical parameters of the 1353 working face strata.

Rock type	Thickness (m)	Bulk density (kg/m^3^)	Bulk modulus (GPa)	Cohesion force (MPa)	Internal friction angle (°)	Tensile strength (MPa)
Coarse sandstone	23.3	2550	13.625	4.32	42	6.23
Fine sandstone	18	2450	10.833	2.75	38	4.01
Mudstone	16	2350	8.266	2.58	33	3.85
Fine sandstone	11.5	2450	10.833	2.75	38	4.01
Coarse sandstone	13.5	2550	13.625	4.32	42	6.23
Siltstone	8.0	2420	11.856	2.83	39	6.91
Silty fine sandstone	11.0	2471	10.833	2.75	38	3.815
Siltstone	6.0	2420	11.856	2.83	39	6.91
Silty fine sandstone	9.0	2471	10.833	2.75	38	3.815
Coal seam	5.3	1376	4.907	1.25	32	0.73
Siltstone	3.3	2420	11.856	2.83	39	6.91
Coal seam	3.5	1376	4.907	1.25	32	0.73
Silty fine sandstone	8.5	2471	10.833	2.75	38	3.815
Siltstone	15.0	2400	12.856	3.83	39	5.06

**Figure 3:**
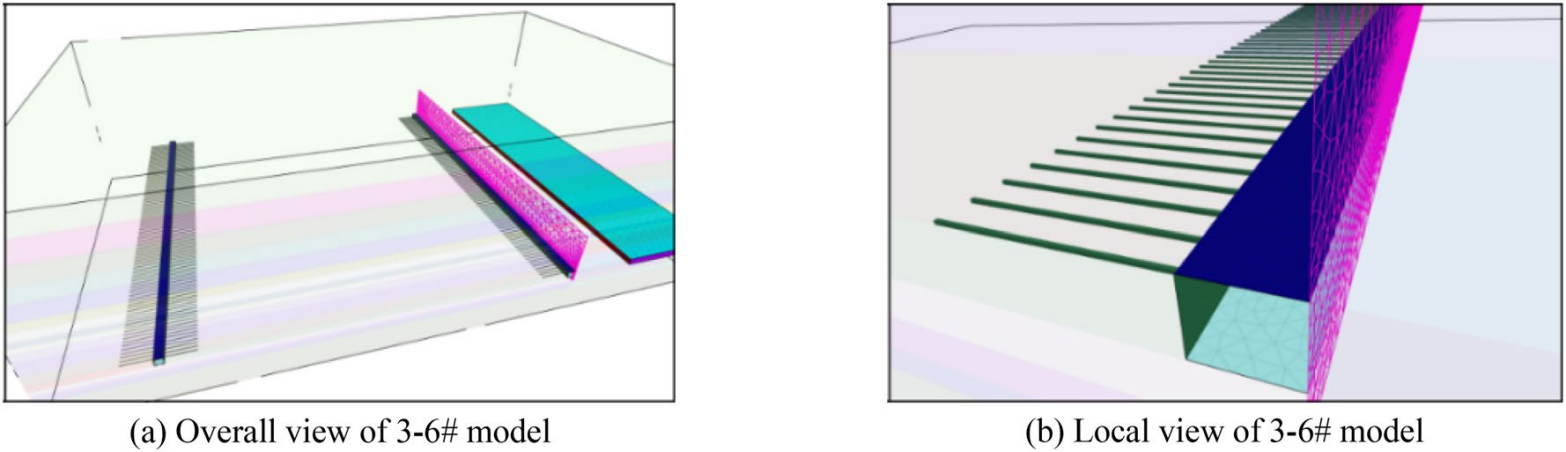
Diagram of 3–6# model.

In order to analyze the pressure relief effect of different schemes, six groups of models were established (Table 3). The two-sided borehole model is shown in Fig. 2, and the broken roof model is shown in Fig. 3. Considering the stress distribution of the working face, the middle position of the working face is selected as the monitoring position.

**Table 3:** Broken top control experimental group.

Scheme (model) number	Feature
1#	No pressure relief measures
2#	Two side borehole pressure relief
3#	A side pressure relief hole, boundary pillar side wall cutting top height of 10 m
4#	A side pressure relief hole, boundary pillar side wall cutting top height of 20 m
5#	A side pressure relief hole, boundary pillar side wall cutting top height of 30 m
6#	A side pressure relief hole, boundary pillar side wall cutting top height of 40 m

## Simulation result analysis

**Figure 4:**
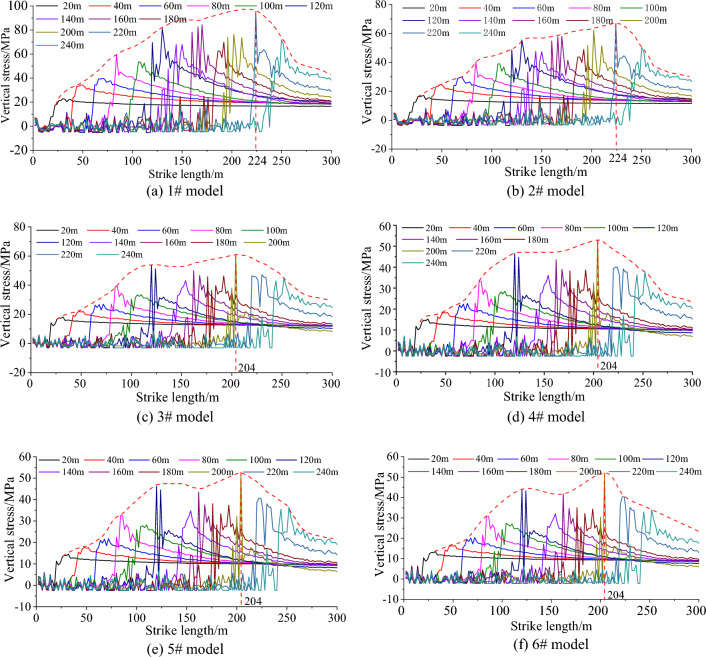
Evolution curve of abutment pressure at middle position of working face.

The change trend of vertical stress along the strike in the middle of different model working faces is shown in Fig. 4.

**Figure 5:**
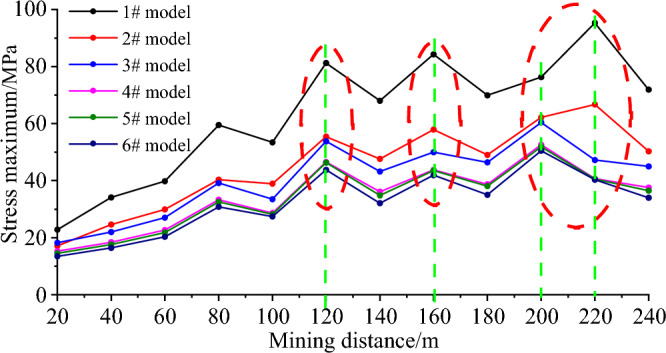
Evolution law of abutment pressure peak during mining process of working face.

The peak stress evolution of the midpoint of the working face under different mining lengths of the 1–6 # model is shown in Fig. 5. As the working face mining, the peak value of the advance abutment pressure gradually increases, but there is an increase–decrease–increase–decrease alternating phenomenon in the intermediate process, which is similar to the periodic fracture of the roof strata above the coal seam. There are obvious local peaks when the working face mines 120 m, 160 m, 200–220 m. Therefore, these three typical positions are selected to compare and analyze the pressure relief effects of different measures.

**Figure 6:**
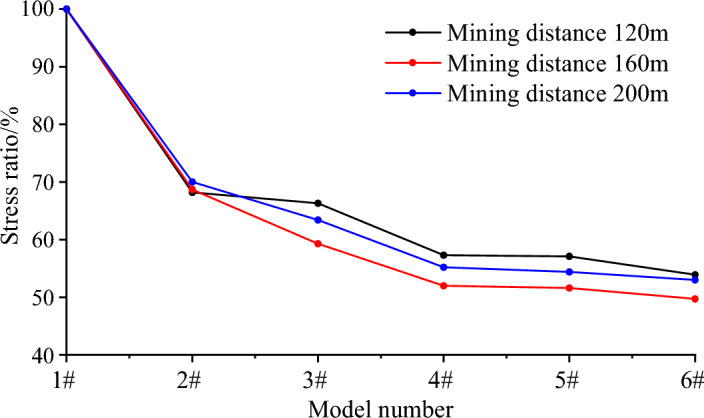
Compared with the stress ratio evolution law of the non-pressure relief model.

The ratio of the peak stress of different models (Table 4) to the peak stress of the non-pressure relief model at the same mining distance is shown in Fig. 6. Peak stresses at the mining lengths of 120 m, 160 m, and 200 m reach 81.2 MPa, 84.3 MPa, and 95.3 MPa, respectively, when the model does not implement pressure relief measures. When the 2# pressure relief scheme is implemented, the peak stress at the corresponding position is significantly lower than that without pressure relief, which is reduced to 55.4 MPa, 57.9 MPa and 66.7 MPa, respectively, which is reduced to 68.2%, 68.7% and 70.0% of the stress of the model without pressure relief. The peak stress is further decreased after the 3# pressure relief scheme is implemented, reaching 53.8 MPa, 50.0 MPa, and 60.4 MPa, which are decreased to 66.3%, 59.3%, and 63.4% of the stress without pressure relief. By comparison, it can be found that the pressure relief scheme of drilling on one side and cutting the roof on the other side is better. This is mainly because the roof cutting and pressure relief reduces the inclined roof span of the working face and effectively releases the energy accumulated in the roadway roof. After adopting the 4# pressure relief scheme, the peak stress is further reduced to 46.5 MPa, 43.8 MPa and 52.6 MPa, which are reduced to 57.3%, 52.0% and 55.2% of the stress without pressure relief, respectively. The stress reduction rate is less than 5% and the peak stress is unaffected by the roof's cutting length when the length of the roof exceeds 20 m. From Fig. 6, it can be seen that the longer the drilling length, the better the pressure relief effect. But when the drilling length exceeds 20 m, the pressure relief effect no longer changes significantly with the drilling length. In summary, the pressure relief scheme of drilling on one side and mining on the other side for 20 m can effectively reduce peak stress, achieve pressure relief effect, greatly reduce the risk of impact on the site, and provide a guarantee for on-site safety production.

**Table 4:** Comparison of abutment pressure peaks (MPa) when the working face mines 120 m, 160 m, 200-220 m.

Mining distance (m)	Model					
	1# model	2# model	3# model	4# model	5# model	6# model
120	81.2	55.4	53.8	46.5	46.3	43.8
160	84.3	57.9	50.0	43.8	43.5	41.9
200–220	95.3	66.7	60.4	52.6	51.8	50.5

**Figure 7:**
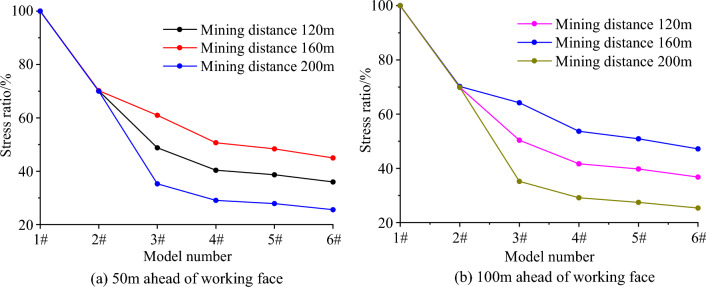
Compared with advance abutment pressure stress ratio evolution law of the non-pressure relief model.

At 50 m and 100 m ahead of the working face, the ratio of the advance abutment pressure stress (Table 5) to the unrelieved advance abutment pressure stress of each model is shown in Fig. 7. Similar to the change of peak stress, when the depth of cutting roof is less than 20 m, the decrease of advance abutment pressure has a significant impact on the length of cutting roof. When the cutting depth is greater than 20 m, the influence of the cutting length on the abutment pressure can be ignored. Considering the stress change and peak stress change law of the advanced working face, the roof cutting depth should be 20 m.

**Table 5:** Abutment pressure of 50 m and 100 m in advance working face under different pressure relief states.

Advance working face distance (m)		Model					
	Mining distance (m)	1#	2#	3#	4#	5#	6#
50	120	40.8	28.6	19.9	16.5	15.8	14.7
	160	34.9	24.5	21.3	17.7	16.9	15.7
	200	34.0	23.8	12.0	9.9	9.5	8.7
100	120	26.6	18.6	13.4	11.1	10.6	9.8
	160	21.8	15.3	14.0	11.7	11.1	10.3
	200	23.6	16.5	8.3	6.9	6.5	6.0

### Mining speed optimization

The failure process of rock is significantly affected by stress path and loading or unloading rate. The mining speed of the working face determines the unloading rate of the surrounding rock, which further has a direct impact on the rock failure process. In the process of rapid mining, that is, under the high unloading rate of surrounding rock, the internal stress of surrounding rock changes abruptly, and a large part of the stress is converted into energy. One part of the energy causes the surrounding rock to be destroyed, and the other part of the energy is converted into the kinetic energy and potential energy of the broken surrounding rock, resulting in the occurrence of pressure bumping. Under the low unloading rate, the energy generated by the sudden change of surrounding rock stress is relatively small. Most of the energy is consumed in the process of surrounding rock crushing, and a small part of the energy is converted into deformation energy of surrounding rock, which can effectively reduce the probability of pressure bumping. ^24,25^ Therefore, reasonable mining speed has important guiding significance in preventing pressure bumping accidents in coal mines. In the mining process of 1353 working face, taking reasonable mining speed, on the one hand, can make the deformation energy in the surrounding rock release slowly, on the other hand, can effectively slow down the overlying strata movement and energy release intensity, effectively avoid the occurrence of pressure bumping.

**Figure 8:**
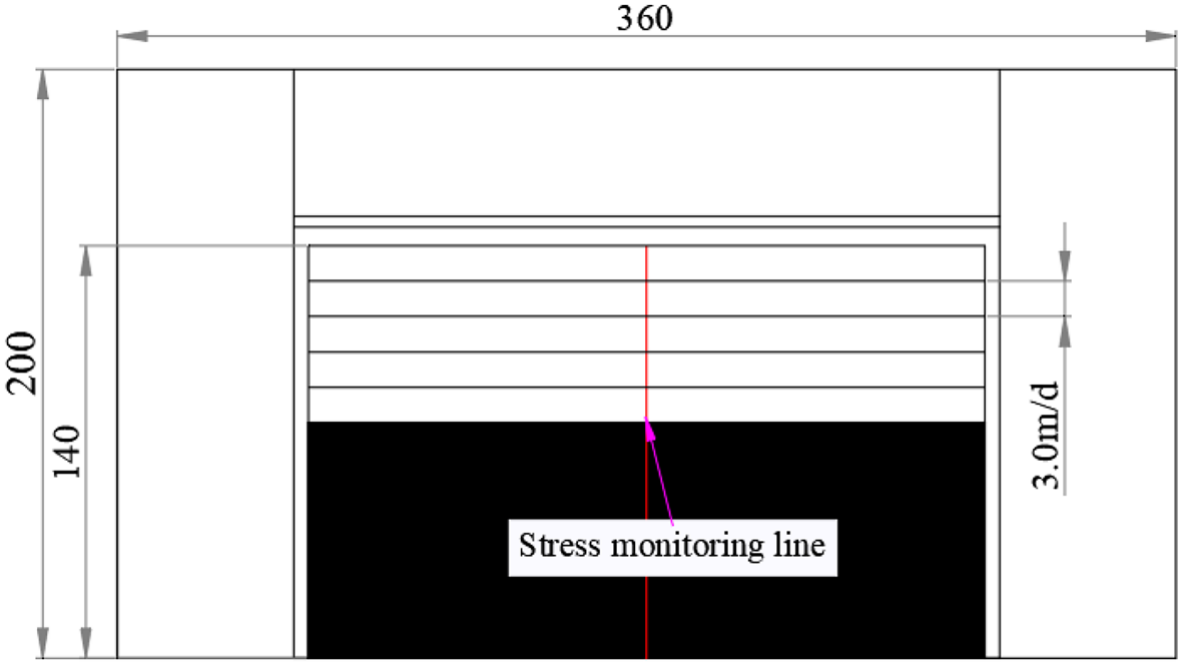
Design model scheme.

Based on the basic situation of the 1353 working face, the design model scheme is shown in Fig. 8. Taking the model boundary as the starting point, the stress monitoring line is arranged in the middle of the roof along the mining direction of the working face. The end point is the open cut, and the measuring line is 140 m long. Four different working face mining speeds of 2.0 m/day, 3.0 m/day, 4.0 m/day and 6.0 m/day were set to explore the influence of working face mining speed on roof stress.

**Figure 9:**
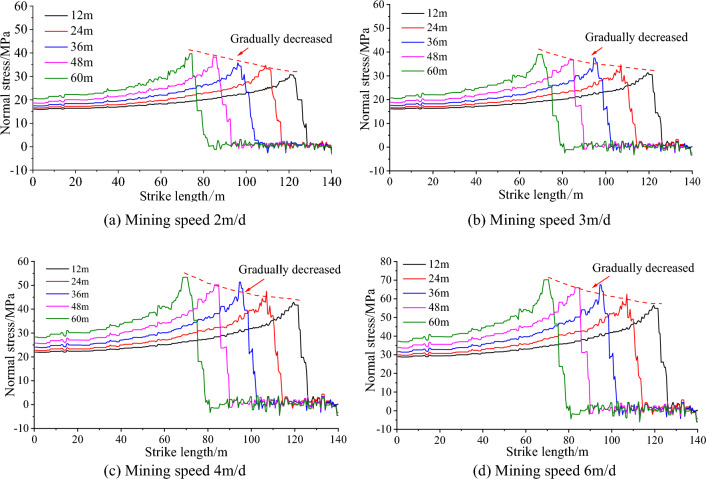
Stress evolution curve of overburden rock.

When the mining speed is 2, 3, 4, 6 m/day, the distribution of vertical stress along the strike length under different mining lengths is shown in Fig. 9. When the mining speed is 2 m/day, the vertical stress of different mining lengths is between 15  and 40 mPa, and the stress value is low; when the mining speed is 6 m/day, the overall stress is high and the stress concentration is obvious. The vertical stress of different mining lengths is between 30 and 70 mPa. With the increase of mining distance, the advanced vertical stress of the working face generally shows an increasing trend, and its increase is mainly affected by the mining speed. The greater the mining speed, the greater the growth rate. The peak velocity stress of different working faces is summarized into Table 5.

**Figure 10:**
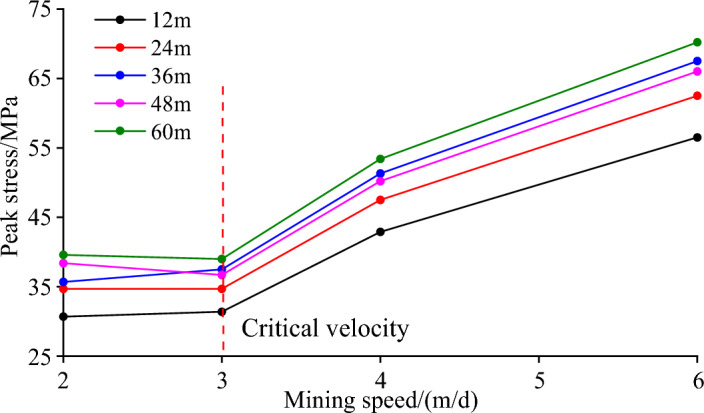
Peak stress at different mining speeds.

It can be seen from Table 6 and Fig. 10 that at the same mining speed, the vertical peak stress is the smallest when the mining distance is 12 m, and the vertical peak stress is the largest when the mining distance is 60 m. The peak stress increases with the increase of the mining distance, but the vertical stress at the mining distance of 36 m increases abnormally, so it is necessary to strengthen monitoring during construction. The peak stress increases with the increase of the mining speed. When the mining speed of the working face is greater than 3.0 m/day, the increase of the peak stress increases sharply. This is mainly due to the dynamic adjustment of overburden structure and stress caused by coal mining unloading, and the stress concentration area will be formed after balance. When the mining speed is too fast, the stress can not be adjusted and transferred, causing the stress concentration area to move to the free surface of the broken area. With the subsequent mining, the dynamic load caused by mining leads to the increase of stress concentration in the stope, and the peak value of stress concentration in coal strata increases. When the mining speed of the working face is less than 3.0 m/day, the stress can be fully redistributed during the continuous excavation, the overburden movement is relatively stable, and the peak stress increases slowly. In view of this, it is determined that the mining speed of the working face does not exceed 3.0 m/day.

**Table 6:** Peak stress of different working face mining speed (MPa).

Mining speed (m/day)	Mining distance				
	12 m	24 m	36 m	48 m	60 m
2.0	30.7	34.7	35.7	38.4	39.6
3.0	31.4	34.7	37.5	36.7	39
4.0	42.9	47.5	51.3	50.2	53.4
6.0	56.5	62.5	67.5	66.0	70.2

### Optimization of stopping line location

The rationality of stopping line location depends on two aspects. One is the risk of stress concentration on pressure bumping caused by the shortening of working face length, and the other is whether the state of roof movement is conducive to the safety of evacuation surface^26,27^.

**Figure 11:**
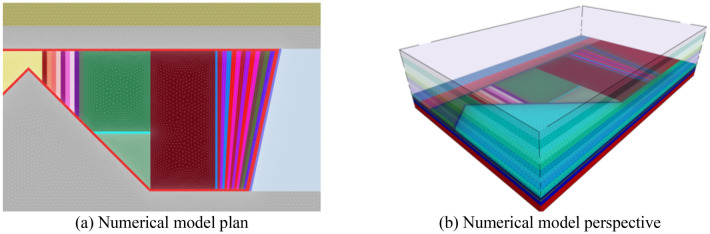
Numerical model diagram.

In order to ensure the safety of the working face after stopping mining, it is necessary to compare the stress evolution curve of the roof at different lengths of the stopping line. The ten numerical models (Fig. 11) with stopping line lengths of 55 m, 65 m, 75 m, 85 m, 95 m, 105 m, 115 m, 125 m, 135 m and 145 m were established for comparative analysis.

**Figure 12:**
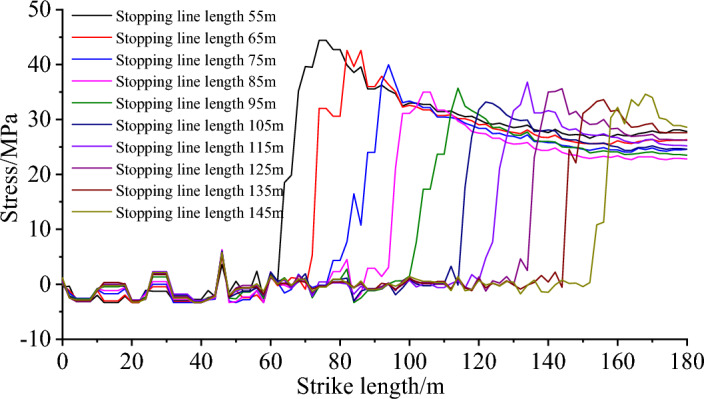
Stress evolution curves of stopping lines with different lengths.

The stress evolution curve of the roof at different lengths of the stopping line is shown in Fig. 12. The diagram shows that a high stress concentration is formed when the roof stress rises sharply in front of the stopping line. The roof stress progressively reduces as it gets farther from the stopping line, eventually returning to the initial state of the rock stress. As the length of the stopping line gradually decreases, and the peak value of the advance abutment pressure gradually increases. It can be seen that the decrease of the length of the stopping line will cause the further concentration of the advance abutment pressure of the working face, which seriously affects the safety production on site.

**Figure 13:**
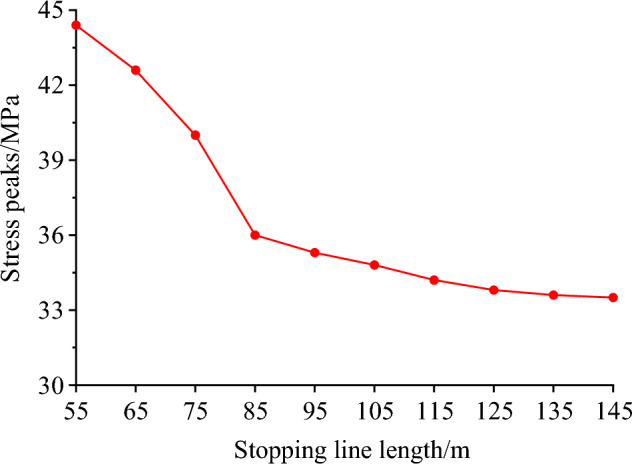
The peak stress evolution trend of stopping line with different lengths.

The evolution trend of peak stress corresponding to different lengths of stopping line is shown in Fig. 13. Statistical analysis shows that when the length of the stopping line is less than 85 m, the peak value of the advance abutment pressure is large, and it is obviously affected by the change of the length of the stopping line. Compared with the model with the stopping line length of 55 m, the peak pressure of the model with the stopping line length of 65 m, 75 m and 85 m decreased by 18.9%, 15.5% and 10.0%, respectively. When the length of the stopping line is less than 85 m, the peak stress is greatly affected by the length of the stopping line, which is not conducive to coal mine safety production. Therefore, the length of the stopping line should not be less than 85 m. Compared with the model with the stopping line length of 85 m, the peak pressure of the model with the stopping line length of 95 m, 105 m, 115 m, 125 m, 135 m and 145 m decreased by 1.9%, 3.3%, 5%, 6.1%, 6.7% and 6.9%, respectively. When the length of the stopping line is more than 85 m, the stress change is small, and the maximum increase is not more than 10%. In summary, when the length of the stopping line is less than 85 m, the stress concentration is high and the peak stress changes significantly with the length of the stopping line. When the length of the stopping line is greater than 85 m, the change of the length of the stopping line has no effect on the stress, and the peak stress fluctuates in a small range. Finally, it is reasonable to determine that the length of the stopping line is greater than 85 m.

### Field application analysis

**Figure 14:**
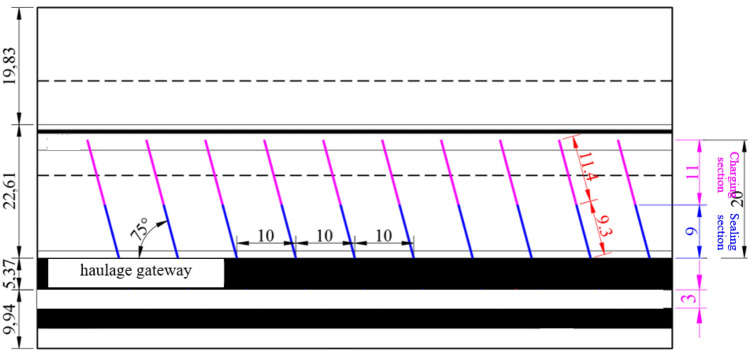
Layout diagram of blasting pressure relief.

According to the numerical simulation analysis and the field situation, it is determined that the 1353 working face of Daizhuang Coalmine adopts the pressure relief method of drilling on one side and cutting the roof 20 m deep on the other side(Fig. 14), and the mining speed of the working face is 3 m/day and the length of the stopping line is 85 m. The pressure relief effect is tracked and observed using the drilling chip method and the microseismic method during the working face mining process.

### Microseismic monitoring

**Figure 15:**
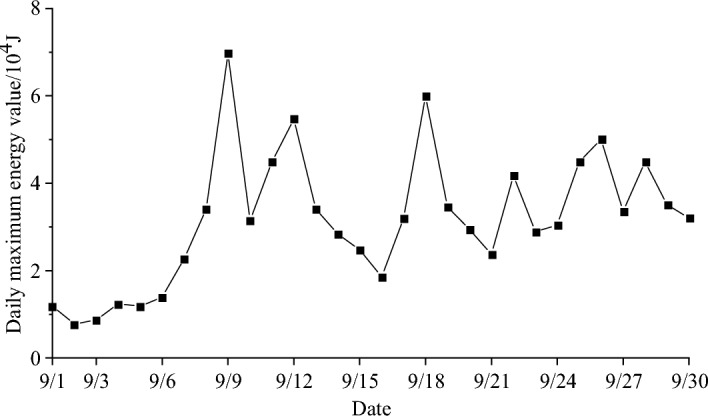
Daily energy peak curve.

The maximum energy curve obtained after one month of pressure relief measures is shown in Fig. 15.

It can be seen from Fig. 15 that after comprehensive treatment measures are taken, the energy level of microseismic events is controlled at 10^4^ J, and most of the microseismic event energy is less than 5 × 10^4^ J. The overall energy change is stable and fluctuates slightly, which meets the needs of safe production in the working face.

### Drilling cuttings monitoring

**Figure 16:**
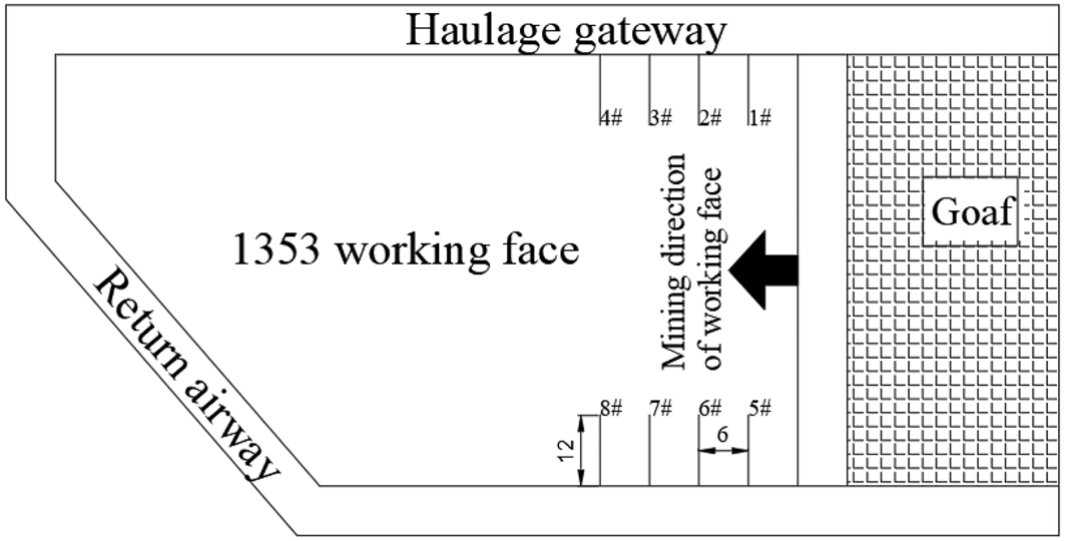
Borehole layout plan.

**Figure 17:**
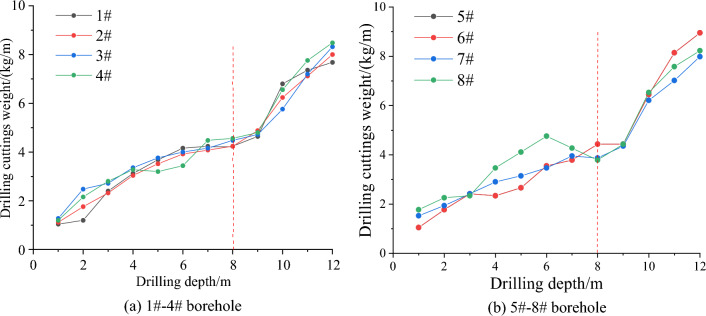
Distribution of pulverized coal.

During the construction process, a hole is drilled every 6 m for monitoring, and a total of 8 holes are drilled, as shown in Fig. 16. During the drilling process, the amount of pulverized coal is weighed and recorded for each 1 m drilling, and whether there are dynamic phenomena such as suction and sticking during the drilling process is recorded. The distribution of pulverized coal is shown in Fig. 17.

Before the implementation of comprehensive treatment measures, the amount of pulverized coal increased rapidly when the hole depth was about 8 m measured by the drilling cuttings method, and there were often phenomena such as sticking, sucking and drilling difficulties. It can be seen that the stress of coal body was concentrated at this place, and there was a possibility of impact danger. After the implementation of comprehensive treatment measures, the amount of pulverized coal is obviously less, and there are few stuck drills and suction drills in the drilling process. These findings indicate that the elastic energy of coal is effectively released and transferred to deeper layers, thereby reducing the risk of dynamic disasters related to impacts.

## Conclusion

1. Aiming at the problem that the side wall of the boundary pillar of the 1353 irregular working face can 't drill, a pressure relief scheme is proposed to drill on one side and cut the roof on the other side. Through numerical simulation and field test, it is verified that the scheme has good pressure relief effect. At the same time, through the comparative analysis of various schemes, the optimal roof breaking height is determined to be 20 m.
2. In response to the problem of impact risk in the 1353 irregular working face, the method of controlling the mining speed is adopted to reduce the impact risk and ensure that the stress in the surrounding rock of the roadway is fully redistributed. The simulation results show that with the increase of mining distance, the increase of overburden stress increases with the increase of mining speed, and the mining speed of 3 m/day is the threshold of overburden stress surge, and then the mining speed of working face is determined to be 3 m/day.
3. Aiming at the problem of stress concentration caused by the decrease of the length of 1353 irregular working face, the safety of the withdrawal surface is ensured by adjusting the length of the stopping line. The simulation results show that the advance abutment pressure is inversely proportional to the length of the stopping line. When the length of the stopping line is less than 85 m, the advance abutment pressure is obviously affected by the length of the stopping line. When the length of the stopping line is greater than 85 m, the advance abutment pressure remains stable, so the length of the stopping line is determined to be 85 m.
4. The practical application proves that the above comprehensive prevention and control measures can effectively improve the surrounding rock state of 1353 working face, which has certain guiding significance for the mining of irregular working face.

## Data Availability

All data generated or analysed during this study are included in this published article [and its supplementary information files].
